# The Complex Relationship Between Veterinarian Mental Health and Client Satisfaction

**DOI:** 10.3389/fvets.2020.00092

**Published:** 2020-02-25

**Authors:** Jennifer L. Perret, Colleen O. Best, Jason B. Coe, Amy L. Greer, Deep K. Khosa, Andria Jones-Bitton

**Affiliations:** Department of Population Medicine, Ontario Veterinary College, University of Guelph, Guelph, ON, Canada

**Keywords:** veterinarian, mental health, client satisfaction, veterinarian-client-patient relationship, burnout, compassion fatigue, resilience, stress

## Abstract

A relatively high risk of poor mental health has been described among Canadian veterinarians, but no published studies have explored the impact that veterinarian mental health may have on veterinary clients and patients. In order to investigate the association between veterinarian mental health and veterinary client satisfaction, veterinarians were randomly sampled and recruited throughout southwestern Ontario, Canada, from November, 2017, through January, 2019. Sixty participating veterinarians completed an enrollment survey that included psychometric scales measuring resilience, perceived stress, anxiety, depression, emotional distress, emotional exhaustion, depersonalization, personal accomplishment, burnout, secondary traumatic stress, and compassion satisfaction. Nine hundred and ninety-five companion animal clients of these veterinarians were recruited in-clinic over 2–3 days and completed a post-appointment survey including the Client Satisfaction Questionnaire. The associations between clients' satisfaction scores (as the outcome variable) and each of the veterinarians' mental health measures (as the explanatory variables) were assessed using separate, multilevel, multivariable linear regression models. The associations between client satisfaction and veterinarian mental health measures were non-linear and complex; in several of the models, relatively higher client satisfaction was unexpectedly associated with poor veterinarian mental health states, while lower client satisfaction was associated with mental health scores suggesting wellness. Given that client satisfaction may impact client adherence to medical recommendations, client loyalty, and business income, the association with veterinarian mental health may have broad implications and warrants further investigation.

## Introduction

Canadian veterinarians are reported to experience relatively poor mental health compared to the general population, including elevated levels of anxiety, depression, and perceived stress, as well as occupation-specific conditions such as burnout and secondary traumatic stress (1). Although possible causes (2, 3), implications for the profession (4), and potential interventions (5) remain important areas for research, to date, little research has been published regarding the occupational impact of veterinarian mental health, and no published studies have explored the effect on patients and clients. This is a gap that warrants investigation, as these stakeholders represent two-thirds of the veterinarian-client-patient relationship.

In human medical professions, the mental health of the caregiver is reportedly associated with caregiver professionalism (6), patient safety (6, 7), and patient satisfaction (6, 7). In the veterinary profession, the unique nature of the veterinarian-patient-client relationship (8) and the broad scope of veterinary general practice [typically including a variety of patient species and ailments (9) as well as variations in the owner-pet bond and client economic factors (10)] can make analysis of patient outcomes challenging. Fortunately, client satisfaction represents an important aspect of healthcare which is more readily measured (11).

The link between client satisfaction and quality of care is complex (12), but veterinary client satisfaction has the potential to influence outcomes for all three members of the veterinarian-client-patient relationship. For example, veterinary client satisfaction has been positively associated with client adherence (8), and intention to adhere (13), to veterinarian recommendations, which is likely to impact patient outcomes. Client adherence to recommendations such as dentistry (8) is also likely to impact clinic income, and indeed, research across customer service industries indicates that client satisfaction and loyalty can have a powerful impact on business revenue (14). Veterinarian mental health and career satisfaction may in turn be impacted by client satisfaction, as relationship-building with clients and patients are among the most rewarding aspects of the veterinary profession (15). Research into the physician-patient relationship suggests that patient satisfaction immediately following an appointment is most strongly linked with the quality of the physician-patient interaction, rather than with the medical outcome (16, 17).

The purpose of this study was to investigate the association of veterinarian mental health measures and appointment-specific veterinary client satisfaction with companion animal appointments in southwestern Ontario, Canada. We hypothesized that veterinary client satisfaction would be positively associated with positive measures of veterinarian mental health (for example, high client satisfaction with high veterinarian resilience), and negatively associated with negative measures of veterinarian mental health (for example, low client satisfaction with high veterinarian burnout).

## Materials and Methods

All surveys were administered in English using Qualtrics software (registered trademark of Qualtrics, Provo, Utah, USA).

### Veterinarians

A target sample size of 60 veterinarian participants was determined based on an a priori estimate of the variability of scores within the population. These estimates were based on the range of scores present for the selected psychometric scales in a recent Canada-wide survey of veterinarians (1), and estimated variation of Client Satisfaction Questionnaire (CSQ) scores based on results from a previous study of veterinarians in Ontario (11). Statistical analyses and sample size calculations were conducted using StataSE 15 software (StataCorp. 2017. *Stata Statistical Software: Release 15*. College Station, TX: StataCorp LLC). A power and sample-size analysis for linear regression was performed using statistical software to calculate sample sizes with an alpha of 5% and a power of 80%. The names, type of practice, and contact details of all active, licensed veterinarians in Ontario were extracted from the College of Veterinarians of Ontario website (18) on October 5th, 2017 (*n* = 4738). Exclusion criteria included: no publicly available contact details; specialist or emergency-only practitioners; non-clinical veterinarians (i.e., government, industry, or academia); mobile, equine, or large-animal-only practitioners; or geographical location >150 km from the Ontario Veterinary College, in Guelph, Ontario, Canada. A random number generator was used to select veterinarians from the final sampling frame for invitation into the study. Selection and recruitment continued simultaneously with data collection until the goal of 60 veterinarian participants was reached.

Selected veterinarians were contacted by the first author and one other graduate-level researcher via email and/or telephone according to clinic or locum contact details available online. Further details on each veterinarian were obtained upon contact, leading to additional exclusions at this stage for reasons such as: having retired or otherwise left practice since the list was first generated; being on parental leave or otherwise not practicing during the period of data collection; working less than one full day per week in-clinic; or having left the study region.

At the time of recruitment, veterinarians were offered an honorarium of $100 for participation, and, based on input from the first four veterinarian participants, were offered an aggregate summary of CSQ results from their clients upon the completion of data collection (provided a minimum of eight clients completed the questionnaire, as required by the research ethics board to maintain client confidentiality). Contact attempts continued until a response or reason for exclusion was obtained for each listed veterinarian. As veterinarian-level factors were of primary interest, rather than clinic-level factors, the selection and participation of multiple veterinarians within a practice was possible and accounted for during analysis.

Within 24 h prior to the start of client recruitment, participating veterinarians completed a survey including demographic and career-related questions as well as the following psychometric scales: the Connor-Davidson Resilience Scale [CD-RISC, (19)], the Perceived Stress Scale [PSS, (20)], the Hospital Anxiety and Depression Scale [HADS, (21)], the Maslach Burnout Inventory—Human Services Survey [MBI-HSS, (22)], and the Professional Quality of Life Scale [ProQOL, (23)].

#### Connor-Davidson Resilience Scale

Resilience, defined in this case as “successful stress-coping ability” [(19), p. 77], was measured using the 10-item CD-RISC. Each item is rated from 0 to 4 according to the participant's experience over the last month, giving a total score range of 0–40. Higher scores represent greater resilience (19). Psychometric properties for the 10-item version are considered excellent, with good internal consistency and construct validity (24). In the absence of resilience-building interventions, CD-RISC scores have been reported to be stable for up to one year (Davidson and Connor, Unpublished). For ease of interpretation, the resilience model was reported with reference to the bottom quartile, interquartile, and upper quartile score categories within this population, as no cut-scores for categories have been reported.

#### Perceived Stress Scale

Perceived stress was defined as the extent to which participants find their lives “unpredictable, uncontrolled, and overloading” [(20), p. 387], and measured using the 10-item version of the PSS. Each item is rated from 0 to 4, giving total scores ranging from 0 to 40. Higher scores represent a higher level of perceived stress (20). A recent review determined that the psychometric properties of the scale are acceptable, and preferable for the 10-item version over the longer or shorter versions (25). The PSS asks participants to consider their thoughts and feelings over the previous month; the test-retest correlation has been reported at 0.85 over two weeks and 0.55 over six weeks (20). For ease of interpretation, the perceived stress model was reported with reference to the bottom quartile, interquartile, and upper quartile score categories within this population, as no cut-scores for categories have been reported.

#### Hospital Anxiety and Depression Scale

Anxiety and depression were measured using the 14-item HADS (21). The HADS is divided into two distinct 7-item subscales and is used to screen for clinical anxiety and/or depression. Each item is rated from 0 to 3, giving total scores ranging from 0 to 21 for each subscale. A higher score indicates a greater likelihood of clinical anxiety or depression. Although previous reviews have suggested excellent construct validity and internal consistency for the separate subscales (26), more recent studies have proposed that the psychometric properties of the scale do not clearly differentiate between anxiety and depression (27). Thus, in addition to the anxiety and depression subscales scores, a total HADS score out of 42 was calculated as a measure of overall emotional distress (27). The HADS asks participants to consider their feelings over the past week (21); however, it has been reported to have excellent test-retest reliability over six months (28).

For ease of interpretation, anxiety and depression subscale models were reported with reference to categories recommended for clinical screening purposes (21): “non-case” (0–7); “possible case” (8–10), or “probable case” (11–17). Overall emotional distress, for which no cut-scores have been previously reported, was classified according to the bottom quartile, interquartile, and upper quartile score categories within this population.

#### Maslach Burnout Inventory—Human Services Survey

The three aspects of burnout were measured using the 22-item MBI-HSS (22). Questions pertain to the feelings of care giving professionals toward their job and the recipients of their care (22), and each item is rated by frequency from 0 to 6. The first subscale, “Emotional Exhaustion,” has 9 items and a total score range of 0–54, with higher scores indicating a higher likelihood of burnout. Emotional exhaustion is defined as “feelings of being emotionally overextended” [(22), p. 15]. The second subscale, “Depersonalization,” consists of 5 items and a total score range of 0–30, again with high scores indicating a higher likelihood of burnout. Depersonalization is defined as “an unfeeling and impersonal response toward recipients of one's service, care… (or) treatment” [(22), p. 15]. The third subscale, “Personal Accomplishment,” is defined as “feelings of competence and successful achievement” [(22), p. 15], has 8 items, and a score range of 0–48. Lower scores in personal accomplishment are suggestive of burnout (22). The MBI is considered the leading measure of burnout, with different versions available for various occupations (22). Of these, the MBI-HSS is the more widely used, and has been found consistently valid and reliable across a range of populations (22). The MBI-HSS has been reported to have good test-retest reliability for at least 2–4 weeks; the depersonalization and personal accomplishment subscales may be reliable for up to one year (22).

For ease of interpretation, subscale models were shown alongside low, average, and high category cut-scores reported for medical occupations (29). Emotional Exhaustion subscale scores were classified as follows: ≤18 as low; 19–26 as average; and ≥27 as high. Depersonalization subscale scores were classified as follows: ≤5 as low, 6–9 as average, and ≥10 as high. Personal Accomplishment subscale scores were classified as follows: ≤33 as low, 34–39 as average, and ≥40 as high (29).

#### Professional Quality of Life Scale

Compassion satisfaction, burnout, and secondary traumatic stress were measured using the 30-item ProQOL, Version 5 (23). The ProQOL is divided into three distinct subscales of 10 items each, with the frequency of each item rated from 1 to 5. Scores for each subscale range from 10 to 50, with higher scores in burnout and secondary traumatic stress indicating negative emotions associated with one's occupation, and higher scores in compassion satisfaction indicating positive emotions associated with care giving, such as “the pleasure you derive from being able to do your work well,” [(23), p. 12]. The ProQOL defines “Burnout” as “the feeling that your efforts make no difference, or… associated with a very high workload or a non-supportive work environment.” [(23), p. 13]. Conversely, “Secondary Traumatic Stress,” previously described as compassion fatigue (30), is specifically related to care giving work and defined as “work related, secondary exposure to people who have experienced extremely or traumatically stressful events” [(23), p. 13]. For the purposes of this study, references to recipients of care (normally, “people”) was replaced with “clients/patients” to reflect the veterinary context. The ProQOL has been widely used in a variety of care giving occupations, and demonstrates good construct validity, with some correlation between the negative subscales (Burnout and Secondary Traumatic Stress) interpreted as shared feeling of work-related distress (23). The ProQOL asks participants to consider their feelings over the past 30 days, and the authors report good test-retest reliability (31).

For ease of interpretation, subscale models were shown alongside low, middle, and high category cut-scores reported for care giving occupations (32). Burnout subscale scores were classified as follows: 10–19 as low, scores 20–26 as average, and 27–50 as high. Secondary Traumatic Stress subscale scores were classified as follows: 10–13 as low, scores 14–20 as average, and 21–50 as high. Compassion Satisfaction subscale scores were classified as follows: 10–33 as low; 34–41 as average; and 42–50 as high (32).

### Clients

Clients were recruited in the reception area of the clinics by the first author or one of two other graduate-level researchers as they arrived for their appointments with the participating veterinarians. Exclusion criteria included: planned euthanasia appointments (for compassionate reasons); clients under the age of majority (18 years in Ontario) or otherwise unable to give consent; and clients who did not speak English. Data were collected from all eligible and consenting clients over the course of two to three full working days with the participating veterinarian, or until the end of a working day once a minimum of 20 of their clients had participated. Client recruitment days with each veterinarian were not necessarily consecutive, yet were scheduled within a three-week period when possible, and within a two-month period in all instances, according to the veterinarian's schedule and preference.

Participating clients completed the CSQ (11), along with questions regarding the reason for their visit, their demographics, and the length of their prior relationship with the veterinarian. Clients were asked to complete the survey in the veterinary clinic immediately following the appointment with the veterinarian; however, if they were unable to do so, they were provided with a secure web link to complete the survey online at a later time. If multiple clients were present during an appointment, they completed only a single survey (jointly, or by the client with the strongest self-reported relationship with the patient). If a client had multiple appointments with a participating veterinarian during the study, only the results of the first completed survey were included in analyses. No questions were mandatory; as such, the number of observations varied by question, as noted.

#### Client Satisfaction Questionnaire

Appointment-specific client satisfaction was assessed using the 15-item CSQ (11). Each item asks about a different aspect of the appointment, and the client rates their satisfaction from “poor” to “could not be better” (1–6), or “unable to assess”. Items left blank or rated “unable to assess” are not included in the average score, so that final scores are an average of only the assessed items (11). Total scores range from 1 to 6. Higher scores indicate a higher degree of client satisfaction with the appointment. A previous study found a difference of 0.8 in CSQ to impact client adherence to dental and/or surgical recommendations by the veterinarian; the mean score among non-adherent clients was 5.0, and the mean score among adherent clients was 5.8 (8). This study result may be used as a guide for interpreting changes in CSQ score reported in the present study.

### Statistical Analyses and Modeling

Linear regression models were constructed to meet the following assumptions (33–35): 1. “continuous predictor variables and outcome variable are linearly related” [p. 592, (33)] or transformed as needed to produce a linear association; 2. continuous outcome variable; 3. independence of covariates; 4. “zero conditional mean error” [p. 593, (33)]; 5. constant variance of errors/homoscedasticity; and 6. uncorrelated errors. Regardless of the distribution of individual variables, models which satisfy these six assumptions are considered robust with regards to estimation of the associations between variables, particularly with sample sizes larger than ~50 (33, 34).

The relationship between CSQ score (as outcome variable) and each continuous independent variable was assessed graphically. If a continuous variable had a non-linear relationship with CSQ score, it was modeled as a polynomial. Polynomial degrees were selected based on graphical assessment of the curvilinear relationship.

The association between each veterinarian mental health measure (as exposure) and client satisfaction score (as outcome) and was assessed separately using multilevel linear regression modeling. Multilevel modeling is a statistical approach used to correct for clustering, nesting, or repeated measures within hierarchical groups (36). In this instance, random intercepts for veterinarian and clinic levels were used to account for the three levels of data: client (lowest level), veterinarian, and clinic (highest level). Given previous associations reported in the literature between healthcare provider age or gender and recipient satisfaction (37, 38), as well as associations between veterinarian mental health measures and age and/or gender [e.g., younger or female veterinarians commonly experience a higher prevalence of poor mental health (1, 39)], a hypothesized interaction between veterinarian age and gender was tested in all models. Additional independent variables were included in the model only if they impacted the association of interest (i.e., if they were confounders of this relationship); therefore, the models were built using forward selection.

Univariable associations with CSQ score were assessed for each possible confounder in a single level linear regression for appointment and client variables, and multilevel linear regression for veterinarian variables. Hypothesized confounders were tested individually in a multilevel, multivariable linear regression model with CSQ score as the outcome to determine the impact on the mental health measure coefficient(s). Confounding was defined as a variable which may affect both veterinarian mental health and client satisfaction, resulting in a change of 30% or more in the coefficient of the mental health variable when added to the model [i.e., impacted the relationship of interest (40–42)]. Client variables such as age, ethnicity, income, education, and veterinary visits in the last year were considered possible confounding variables in proxy for demographics of the region/clinic clientele. Appointment variables such as pet species, number of pets present, and type of appointment were also considered as proxy for typical clinic clientele. The length of the relationship between the client and the veterinarian was considered a proxy for the length of time the veterinarian had practiced in the clinic. Veterinarian clinic role and veterinarian ethnicity were also considered as possible confounding variables.

Multilevel, multivariable linear regression models of the association between CSQ score and each mental health measure were generated by including the association of interest, veterinarian gender, veterinarian age, and all confounders, as well as random effects at the veterinarian and clinic levels. Model fit was assessed using the likelihood ratio test (LRT; the likelihood that the multilevel model had a better fit than a single level linear regression model) and the Wald χ^2^ test (the likelihood that all of the coefficients in the model are equal to zero), as well as a graphical analysis of the best linear unbiased predictors [(40), BLUPs]. The distribution of the BLUPs indicates the overall fit of a multilevel model (i.e., a normal distribution indicates a good fit). The impact of any outliers at the veterinarian level was assessed by running the models without outlying observations. Statistical significance was defined as *p* < 0.05.

For ease of interpretation, we calculated the model-estimated difference between the minimum and maximum CSQ scores (within a 95% confidence interval) associated with each mental health measure in this population.

## Results

### Veterinarian Participants

A total of 2,234 veterinarians met the initial inclusion process. This involved the random selection of 472 veterinarians overall, of which 257 declined participation, 149 met one or more exclusion criteria, and five who had been contacted but had not responded at the time of the conclusion of the study. The most common reasons given for declining participation were as follows: lack of interest; feeling too busy; and feeling self-conscious (the project also involved video-recording of appointments for a different study). Sixty-one veterinarians agreed to participate, resulting in an acceptance of 19.2% (61 out of 318 who were eligible for inclusion and provided a response). One veterinarian agreed to participate but was not scheduled for study dates prior to the conclusion of the project, as the sample size goal had already been reached. Data were collected from 60 veterinarians at 55 different clinics.

Fifty-six (93%) participants completed their study dates within a three-week period. Fifty-seven (95%) participants practiced small animal medicine only; the remainder practiced mixed (both large and small) animal medicine. Twelve participants reported part-time hours (<30 h per week), 23 reported working an average of 30–40 h per week, and 25 reported working more than 40 h per week. Further characteristics of participating veterinarians are presented in Table 1, as well as the univariable associations with CSQ score. Mental health measure results for participating veterinarians are listed in Table 2.

**Table 1:** Description of participating veterinarians (n = 60) and predicted impact on Client Satisfaction Questionnaire (CSQ) score in univariable linear regression.

**Veterinarian characteristics**				**Predicted impact on CSQ score (CI)**	***P***
Age in years		Mean = 47 (range, 29–64)		−0.001 (−0.006 to 0.005)	0.83
Year of graduation from veterinary school		Mean = 1997 (range, 1979–2015)		*n/a; correlation with age: 97.1%*	
		*n*	%		
Gender	Female	39	65.0	*Referent*	
	Male	21	35.0	−0.17 (−0.29 to −0.060)	0.003^*^
Ethnicity	Caucasian	44	73.3	*Referent*	
	Other^†^	16	26.7	−0.13 (−0.26 to −0.001)	0.048^*^
Clinic role	Associate or locum	24	40.0	*Referent*	
	Owner	36	60.0	−0.055 (−0.17 to 0.059)	0.34

* *Variables with a significant (P < 0.05) association with CSQ score in univariable linear regression*.

† *Ethnicity category options were collapsed into “other” due to small cell size*.

**Table 2:** Mental health outcome scores of participating veterinarians (*n* = 60) and predicted impact on Client Satisfaction Questionnaire (CSQ) score (*n* = 995) in multilevel, univariable linear regression.

**Scale or subscale**	**Mean** **(Median)**	**Standard deviation**	**IQR** **(Range)**	**Cronbach's alpha**	**Polynomial degree**	**Predicted impact on CSQ score (CI)**	***P***
Connor-Davidson Resilience	31.1 (30.5)	5.7	28.0–35.0 (19–40)	0.89	1st	−0.78 (−1.51 to −0.054)	0.035^*^
					2nd	0.028 (0.003 to 0.053)	0.029^*^
					3rd	−3.2e^−4^ (−0.001 to −3.9e^−5^)	0.025^*^
Perceived stress	14.2 (14.0)	6.8	9.0–17.5 (2–31)	0.90	1^st^	0.038 (−0.054 to 0.13)	0.42
					2nd	−0.004 (−0.010 to 0.002)	0.21
					3rd	1.0e^−4^ (−2.5e^−5^ to 2.3e^−4^)	0.11
*HADS*^‡^ Anxiety	6.6 (6.0)	4.0	3.5–9.0 (0–17)	0.86	1st	−0.034 (−0.08 to −0.010)	0.13
					2nd	0.0021 (0.001 to 0.005)	0.11
*HADS*^‡^ Depression	3.7 (3.0)	3.2	1.0–5.0 (0–16)	0.77	1st	−0.049 (−0.091 to −0.008)	0.019^*^
					2^nd^	0.003 (−1.2e^−4^ to 0.007)	0.059
*HADS*^‡^ Emotional distress	10.3 (9.0)	6.4	5.5–14.5 (0–21)	0.87	1st	−0.028 (−0.058 to 0.001)	0.060
					2^nd^	0.001 (1.4e^−4^ to 0.002)	0.085
*MBI-HSS*^§^ Emotional exhaustion	21.4 (19.5)	13.0	10.5–32.0 (0–53)	0.94	1st	0.087 (0.014 to 0.16)	0.019^*^
					2nd	−0.007 (−0.012 to −0.002)	0.010^*^
					3rd	1.9e^−4^ (3.7e^−5^ to 3.4e^−4^)	0.014^*^
					4th	−1.6e^−6^ (−3.0e^−6^ to −1.7e^−7^)	0.028^*^
*MBI-HSS*^§^ Depersonalization	7.7 (6.0)	5.8	3.0–11.0 (0–23)	0.81	1^st^	0.046 (−0.027 to 0.12)	0.22
					2nd	−0.008 (−0.016 to 1.3e^−4^)	0.054
					3rd	2.8e^−4^ (4.3e^−5^ to 0.001)	0.021^*^
*MBI-HSS*^§^ Personal accomplishment	38.8 (39.5)	6.6	35.0–44.0 (21–48)	0.77	1st	0.011 (0.003 to 0.019)	0.010^*^
*ProQOL*^||^ Burnout	22.3 (21.0)	6.1	18.0–26.5 (10–37)	0.81	1^st^	1.64 (0.69 to 2.59)	0.001^*^
					2nd	−0.11 (−0.18 to −0.047)	0.001^*^
					3rd	0.003 (0.001 to 0.005)	0.001^*^
					4th	−3.3e^−5^ (−5.3e^−5^ to −1.2e^−5^)	0.002^*^
*ProQOL*^||^ Secondary traumatic stress	22.4 (21.5)	6.2	18.0–25.5 (13–39)	0.83	1^st^	1.64 (0.30 to 2.97)	0.016^*^
					2nd	−0.10 (−0.19 to −0.019)	0.017^*^
					3rd	0.003 (4.9e^−5^ to 0.005)	0.018^*^
					4th	−2.7e^−5^ (−4.9e^−5^ to −4.3e^−6^)	0.020^*^
*ProQOL*^||^ Compassion satisfaction	40.9 (41.0)	6.1	37.0–46.0 (26–50)	0.92	1st	−0.094 (−0.20 to 0.012)	0.083
					2nd	0.001 (−0.000 to 0.003)	0.069

* *Variables with a significant (P < 0.05) association with CSQ score in univariable linear regression*.

‡ *Hospital Anxiety and Depression Scale*.

§ *Maslach Burnout Inventory—Human Services Survey*.

|| *Professional Quality of Life*.

### Client Participants

A total of 1183 clients were asked to participate in the study. Of these, 84.1% (*n* = 995) completed the CSQ, giving a mean of 16.6 CSQ scores per veterinarian. Of those who completed the CSQ, 85.1% (847/995) completed it prior to leaving the clinic, with the remainder completing it online at a later time. CSQ scores were positively skewed, with a median of 5.00 and a mean of 5.20 (standard deviation = 0.68; range = 2.33–6.00; interquartile range = 4.87–5.91). The mean CSQ score did not differ significantly (*t* = −1.10; *p* = 0.27) between clients who completed all items in the CSQ (5.19; *n* = 687), and those who left some items blank or replied “Unable to assess” on some items (5.24; *n* = 308). CSQ scores for questionnaires completed prior to leaving the clinic were significantly higher than those completed online (4.92; *t* = 4.63; *p* < 0.001) but the time between appointment and completion of the CSQ could not be reliably determined, and these results were not excluded. Characteristics of participating clients are presented in Table 3, as well as univariable associations with CSQ score.

**Table 3:** Description of clients who completed the Client Satisfaction Questionnaire (CSQ; *n* = 995) and predicted impact of client variables on CSQ score in univariable linear regression.

**Client characteristics**				**Predicted impact on CSQ score (CI)**	***P***
Age in years (*n* = 929)	Mean = 45.7 (range, 18–84)			0.004 (<0.001 to 0.007)	0.013^*^
Years client has known the veterinarian (*n* = 954)	Mean = 6.1 (range, 0–50)			0.016 (0.010 to 0.022)	<0.001^*^
Veterinary visits in the last year (*n* = 936)	Mean = 4.4 (range, 0–365)			0.002 (−0.001 to 0.006)	0.17
		*n*	%		
Gender (*n* = 967)	Female	713	73.7	*Referent*	
	Male	253	26.2	−0.027 (−0.12 to 0.070)	0.58
	Non-binary	1	0.1	0.58 (−0.74 to 1.91)	0.39
Ethnicity (*n* = 945)	Caucasian	831	87.9	*Referent*	
	Asian	51	5.4	−0.32 (−0.51 to −0.13)	0.001^*^
	Hispanic	15	1.6	−0.22 (−0.57 to 0.12)	0.20
	African American	15	1.6	−0.11 (−0.45 to 0.23)	0.53
	First nation/native^†^	5	0.5%		
	Pacific Islander^†^	4	0.4		
	Middle Eastern^†^	3	0.3		
	Other	21	2.2	−0.075 (−0.31 to 0.16)	0.53
Annual income (*n* = 840)	Less than $20,000	33	3.9%	*Referent*	
	$20,000–$34,999	60	7.1	0.063 (−0.22 to 0.35)	0.66
	$35,000–$49,999	85	10.1	−0.040 (−0.31 to 0.23)	0.77
	$50,000–$74,999	145	17.3	0.19 (−0.066 to 0.44)	0.15
	$75,000–$99,999	142	16.9	0.20 (−0.055 to 0.45)	0.13
	$100,000–$149,999	180	21.4	0.12 (−0.13 to 0.37)	0.34
	More than $150,000	195	23.2	0.17 (−0.073 to 0.42)	0.17
Highest level of education achieved(*n* = 957)	Less than high school	20	2.1	*Referent*	
	High school diploma or equivalent	121	12.6	0.14 (−0.18 to 0.46)	0.39
	Some college or university	156	16.3%	0.094 (−0.22 to 0.41)	0.56
	College diploma	282	29.5	0.026 (−0.28 to 0.33)	0.87
	Bachelor degree	214	22.4	0.004 (−0.30 to 0.31)	0.98
	Graduate degree	106	11.1	0.067 (−0.25 to 0.39)	0.68
	Professional degree	58	6.1	0.12 (−0.22 to 0.46)	0.50

* *Variables with a significant (P < 0.05) association with CSQ score in univariable linear regression*.

† *Due to small cell size, First Nation/Native, Pacific Islander, and Middle Eastern ethnicities were collapsed into the “other” ethnicity category during modeling (total n = 33)*.

Characteristics of appointments are listed in Table 4, as well as univariable associations with CSQ score. Veterinarian participants reported that 4.6% (20/437) of appointments which had been scheduled as “routine wellness or annual exam” should have been booked as a problem visit.

**Table 4:** Description of appointments and predicted impact of appointment variables on Client Satisfaction Questionnaire (CSQ) score in univariable linear regression.

**Appointment characteristic**				**Predicted impact on CSQ score (CI)**	***P***
Number of pets present (*n* = 995)	Mean = 1.08 (range, 0–5)			−0.012 (−0.14 to 0.11)	0.86
		*n*	%		
Species of pet(s) present (*n* = 987)	Dog(s) only	749	75.9	*Referent*	
	Cat(s) only	209	21.2	−0.072 (−0.18 to 0.032)	0.17
	Rabbit(s) only	10	1.0	−0.53 (−0.95 to −0.11)	0.014^*^
	Dog(s) and cat(s)	7	0.7	0.15 (−0.24 to 0.53)	0.46
	Other (i.e., bird, reptile, rat)	12	1.2	−0.11 (−0.61 to 0.39)	0.67
Reason for appointment (*n* = 986)	Routine wellness/annual exam	451	45.7	*Referent*	
	Initial problem (i.e., injury, illness)	317	32.2%	−0.066 (−0.16 to 0.032)	0.19
	Follow-up or recheck	201	20.4	−0.006 (−0.12 to 0.11)	0.92
	Multiple reasons	17	1.7	−0.022 (−0.35 to 0.31)	0.89

* *Variables with a significant (P < 0.05) association with CSQ score in univariable linear regression*.

### Multilevel, Multivariable Linear Regression Models of Client Satisfaction

**Figure 1:**
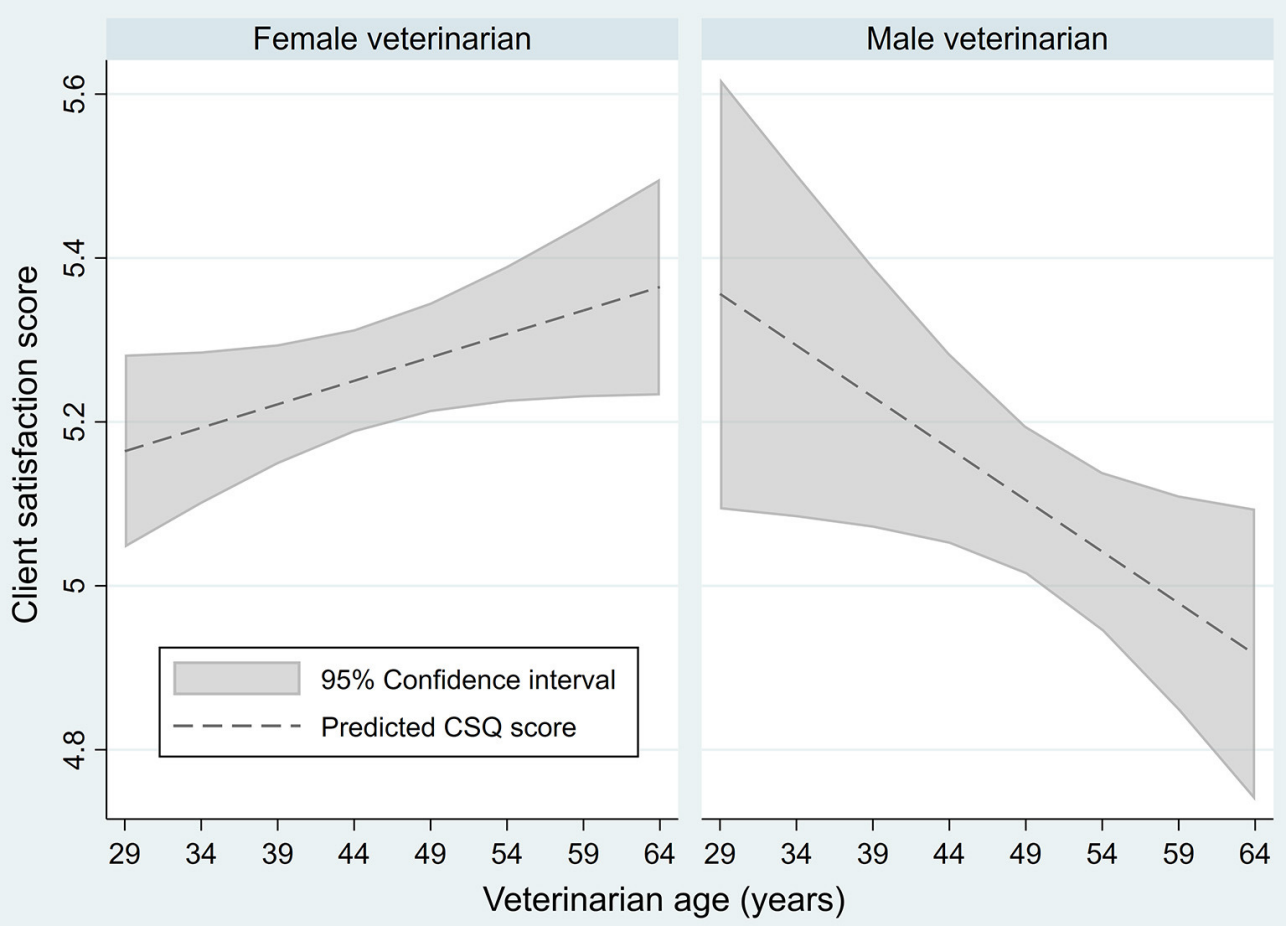
Predicted association between Client Satisfaction Questionnaire scores of veterinary clients (*n* = 995) and veterinarian age and gender (*n* = 60) in a univariable, multilevel linear regression.

The interaction between veterinarian gender and age had a significant association with CSQ score in multilevel, univariable analysis (*p* = 0.006; Figure 1), as well as all multilevel, multivariable models. For female veterinarians, CSQ score was positively associated with age; i.e., higher scores were associated with older female veterinarians. For male veterinarians, CSQ score was negatively associated with age; i.e., lower scores were associated with older male veterinarians.

#### Connor-Davidson Resilience Scale

The relationship between CSQ score and CD-RISC score was not linear and was best fit with a third-order polynomial of the CD-RISC score (Table 2).

In multilevel, multivariable analysis, the relationship between CSQ score and veterinarian CD-RISC score was confounded by client ethnicity and client income. The interaction between veterinarian gender and age was significant in the model but had little effect on the relationship between CSQ score and CD-RISC score. The full model had a significant fit relative to a null model (Wald χ^2^ = 45.18, *p* < 0.001), but the LRT was not significant (${\bar{\chi }}^{2}$ = 1.85, *p* = 0.40). The BLUPs had a relatively normal distribution on graphical analysis with four outliers at the veterinarian level. When the model was run without the outlying observations, the association of CD-RISC and CSQ scores did not change substantially but increased modestly in statistical significance. The final model presented here includes the data from all 60 veterinarians and 823 clients.

**Figure 2:**
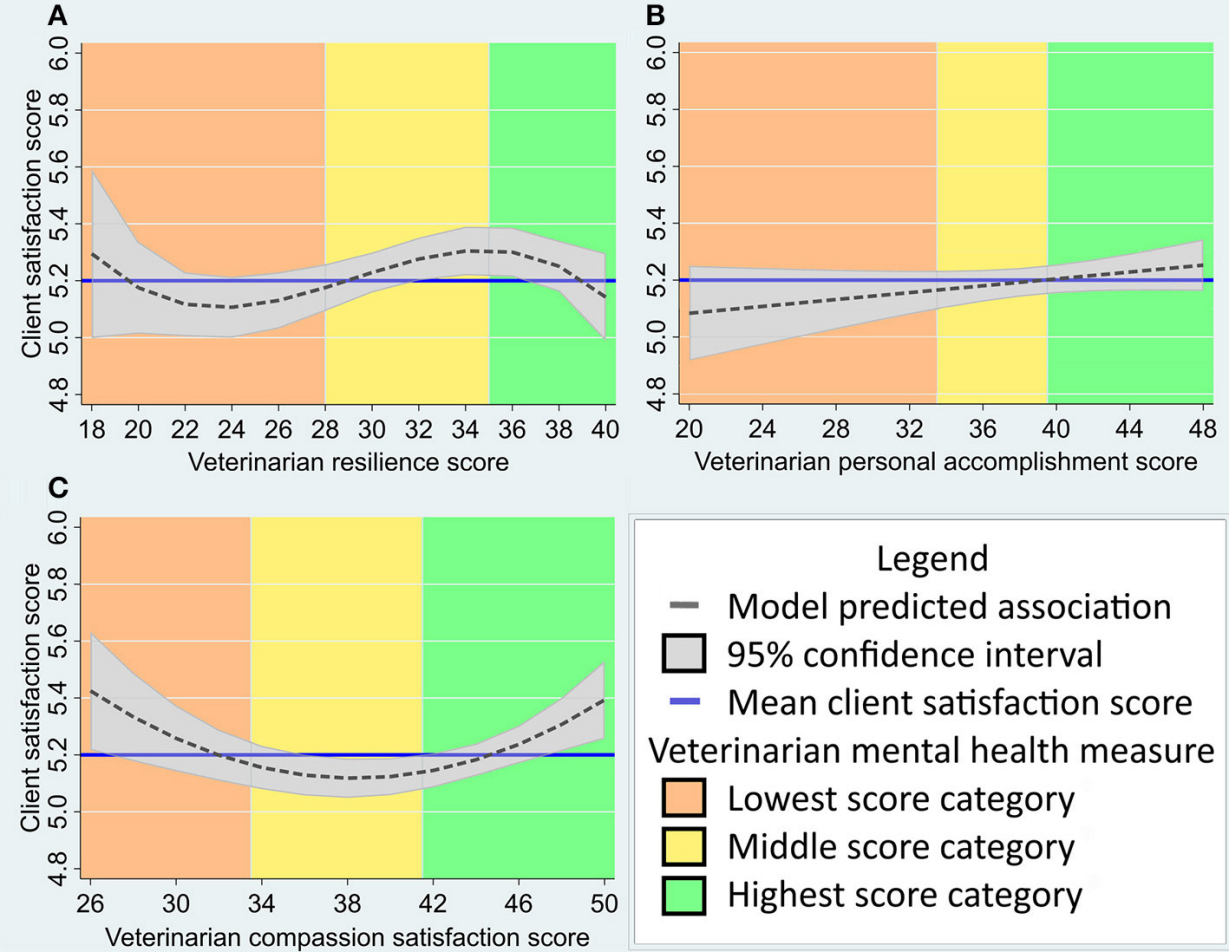
**(A–C)** Predicted association between veterinary client satisfaction (as measured by the Client Satisfaction Questionnaire, *n* varies from 823 to 995 as described in results) and veterinarian mental health measures (*n* = 60) in a multivariable, multilevel linear regression model. Veterinarian mental health measures include: **(A)** Connor-Davidson Resilience Scale score; **(B)** MBI-HSS Personal Accomplishment subscale score; and **(C)** ProQOL Compassion Satisfaction subscale score. Mean client satisfaction score and mental health measure categories (as described in methods) are provided for reference. The Y-axis has been standardized to include the interquartile score range for this population (4.9–5.9) and the X-axis has been adjusted for the range of scores seen in this population.

Overall, the multilevel, multivariable model estimated an association of higher CSQ scores with veterinarian CD-RISC scores in the upper quartile, and lower CSQ scores with veterinarian CD-RISC scores in the bottom quartile (Figure 2A). The model estimated a maximum difference of ~0.6 on the CSQ scale associated with veterinarian resilience scores in this population.

#### Perceived Stress Scale

The relationship between CSQ score and PSS score was not linear and was best fit with a third-order polynomial of the PSS score (Table 2).

In multilevel, multivariable analysis, the association between CSQ score and veterinarian PSS score was confounded by veterinarian clinic role, client ethnicity, and client income. The interaction between veterinarian gender and age was significant in the model and reduced the magnitude of the association between CSQ score and PSS score. The full model had a significant fit relative to a null model (Wald χ^2^ = 50.27, *p* < 0.001), but the LRT was not significant (${\bar{\chi }}^{2}$ = 1.19, *p* = 0.55). The BLUPs had a relatively normal distribution on graphical analysis with two outliers at the veterinarian level. The change to PSS coefficients was minimal when these outliers were excluded, though 95% confidence intervals were smaller. The final model presented here includes the data from all 60 veterinarians and 823 clients.

**Figure 3:**
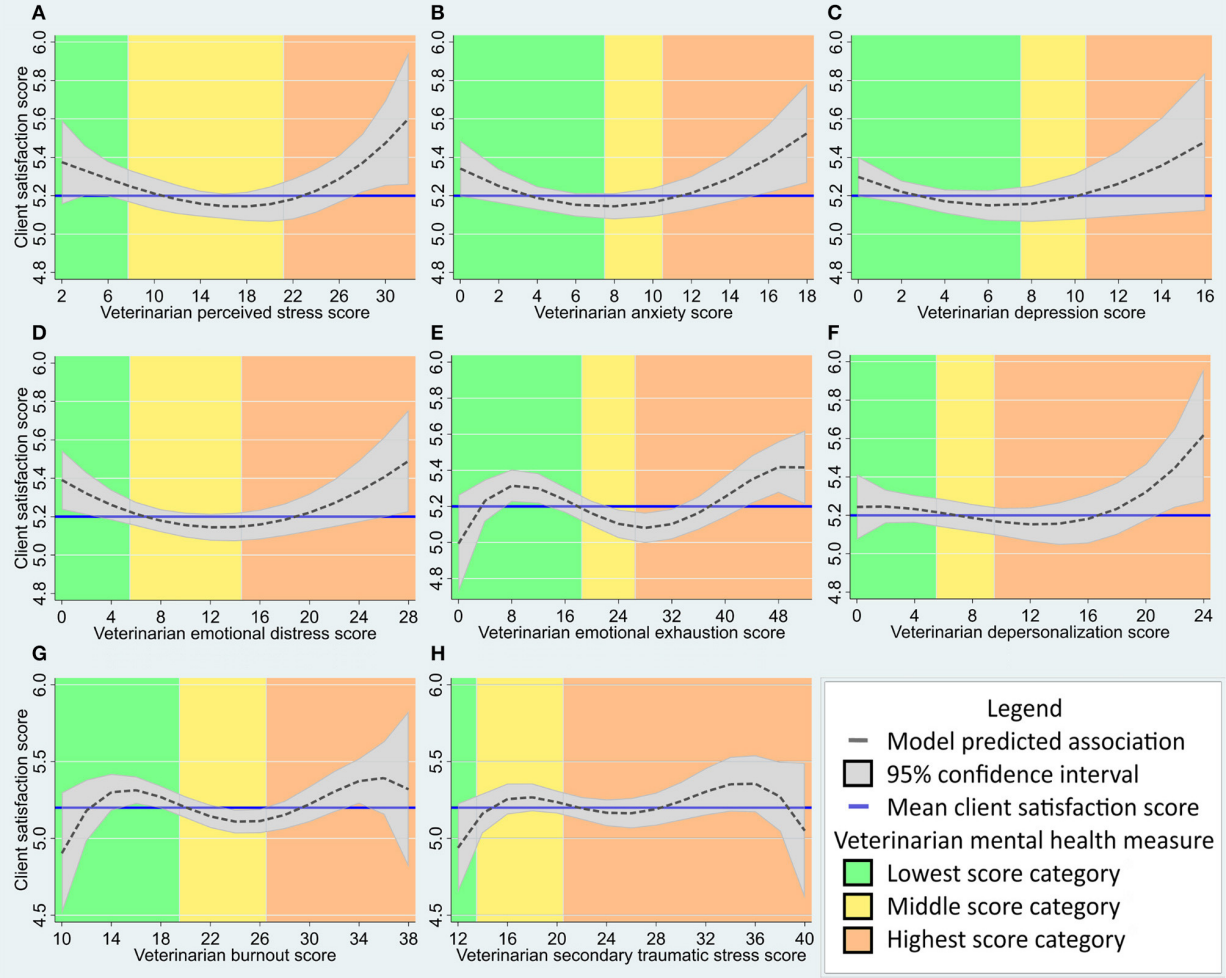
**(A–H)** Predicted association between veterinary client satisfaction (as measured by the Client Satisfaction Questionnaire, *n* varies from 800 to 995 as described in results) and veterinarian mental health measures (*n* = 60) in a multivariable, multilevel linear regression model. Veterinarian mental health measures include: **(A)** Perceived Stress Scale score; **(B)** Hospital Anxiety and Depression Scale (HADS) anxiety subscale score; **(C)** HADS depression subscale score; **(D)** HADS emotional distress score; **(E)** Maslach Burnout Inventory—Human Services Survey (MBI-HSS) Emotional Exhaustion subscale score; **(F)** MBI-HSS Depersonalization subscale score; **(G)** Professional Quality of Life (ProQOL) Burnout subscale score; and **(H)** ProQOL Secondary Traumatic Stress subscale score. Mean client satisfaction score and mental health measure categories (as described in methods) are provided for reference. The client satisfaction score axis has been standardized to include the interquartile score range for this population (4.9–5.9) and the X-axis has been adjusted for the range of scores seen in this population.

Overall, the multilevel, multivariable model estimated an association of higher CSQ scores with veterinarian PSS scores in the upper quartile, relative to PSS scores in the interquartile range or bottom quartile (Figure 3A). The model estimated a maximum difference of ~0.9 on the CSQ scale associated with veterinarian perceived stress scores in this population.

#### HADS Anxiety Subscale

The relationship between CSQ score and HADS anxiety subscale score was not linear and a second-order polynomial provided a marginally better fit than a third-order polynomial (Table 2).

In multilevel, multivariable analysis, the interaction between veterinarian gender and age was significant and increased both the magnitude and significance of the association between CSQ score and anxiety score. No additional independent variables met the criteria for confounding. The full model had a significant fit relative to a null model (Wald χ^2^ = 28.7, *p* < 0.001), but the LRT was not significant (${\bar{\chi }}^{2}$ = 3.91, *p* = 0.14). The BLUPs had a relatively normal distribution on graphical analysis with two outliers at the veterinarian level. When the model was run without the outlying observations, there was minimal change to the coefficients of anxiety score. The final model presented here includes the data from all 60 veterinarians and 995 clients.

Overall, the multilevel, multivariable model estimated an association of higher CSQ scores with veterinarian anxiety scores at the upper and lower ends of the range, and lower CSQ scores with veterinarian anxiety scores in the mid-range (Figure 3B). The model estimated a maximum difference of ~0.7 on the CSQ scale associated with veterinarian anxiety scores in this population.

#### HADS Depression Subscale

The relationship between CSQ score and HADS depression subscale score was not linear and a second-order polynomial provided the best fit (Table 2).

In multilevel, multivariable analysis, the association between CSQ score and veterinarian depression score was confounded by the number of years the client had known the veterinarian, client income category, and client education category. The interaction between veterinarian gender and age was significant in the model and reduced the magnitude and significance of the association between CSQ score and depression score. The full model had a significant fit relative to a null model (Wald χ^2^ = 63.69, *p* < 0.001), but the LRT was not significant (${\bar{\chi }}^{2}$ = 2.22, *p* = 0.33). The BLUPs had a relatively normal distribution on graphical analysis with two outliers at the veterinarian level. When the model was run without the outlying observations, the magnitude of the depression score coefficients was reduced. The final model presented here includes the data from all 60 veterinarians and 819 clients.

Overall, the multilevel, multivariable model estimated an association of higher CSQ scores with veterinarian depression scores at the upper and lower ends of the range, and greater variation of CSQ scores associated with higher veterinarian depression scores (Figure 3C). The model estimated a maximum difference of ~0.75 on the CSQ scale associated with veterinarian depression scores in this population.

#### HADS Total Score—Emotional Distress

The relationship between CSQ score and HADS emotional distress score (i.e., HADS total score) was not linear and a second-order polynomial provided the best fit (Table 2).

In multilevel, multivariable analysis, the association between CSQ score and veterinarian emotional distress score was confounded by client income category. The interaction between veterinarian gender and age was significant in the model and increased the significance of the association between CSQ score and emotional distress score. The full model had a significant fit relative to a null model (Wald χ^2^ = 41.07, *p* < 0.001), but the LRT was not significant (${\bar{\chi }}^{2}$ = 2.02, *p* = 0.36). The BLUPs had a relatively normal distribution on graphical analysis with three outliers at the veterinarian level. When the model was run without the outlying observations, the magnitude and significance of the emotional distress score coefficients was reduced. The final model presented here includes the data from all 60 veterinarians and 840 clients.

Overall, the multilevel, multivariable model estimated an association of higher CSQ scores with veterinarian emotional distress scores at the upper and lower ends of the range, and lower CSQ scores with veterinarian emotional distress scores in the mid-range (Figure 3D). The model estimated a maximum difference of ~0.7 on the CSQ scale associated with veterinarian emotional distress scores in this population.

#### MBI-HSS Emotional Exhaustion Subscale

The relationship between CSQ score and MBI-HSS emotional exhaustion subscale score was not linear and a fourth-order polynomial provided the best fit (Table 2).

In multilevel, multivariable analysis, the association between CSQ score and veterinarian emotional exhaustion score was confounded by client ethnicity category and client education category. The interaction between veterinarian gender and age was significant in the model and modestly decreased the magnitude and significance of the emotional exhaustion coefficients. The full model had a significant fit relative to a null model (Wald χ^2^ = 48.7, *p* < 0.001), but the LRT was not significant (${\bar{\chi }}^{2}$ = 0.15, *p* = 0.93). The BLUPs had a normal distribution on graphical analysis with three outliers at the veterinarian level. When the model was run without the outlying observations, the magnitude and significance of the emotional exhaustion score coefficients was marginally reduced. The final model presented here includes the data from all 60 veterinarians and 995 clients.

Overall, the multilevel, multivariable model estimated an association of higher CSQ scores with veterinarian emotional exhaustion at the upper and lower ends of the range relative to the mid-range, with the greatest variation in CSQ scores associated with veterinarian emotional exhaustion close to zero (Figure 3E). The model estimated a maximum difference of ~0.9 on the CSQ scale associated with veterinarian emotional exhaustion scores in this population.

#### MBI-HSS Depersonalization Subscale

The relationship between CSQ score and MBI-HSS depersonalization subscale score was not linear and a third-order polynomial provided the best fit (Table 2).

In multilevel, multivariable analysis, the association between CSQ score and veterinarian depersonalization score was confounded by client age, client income category, client education category, and the number of years the client had known the veterinarian. The interaction between veterinarian gender and age was significant in the model and modestly increased the significance of the depersonalization coefficients. The full model had a significant fit relative to a null model (Wald χ^2^ = 62.05, *p* < 0.001), but the LRT was not significant (${\bar{\chi }}^{2}$ = 1.21, *p* = 0.55). The BLUPs had a relatively normal distribution on graphical analysis with four outliers at the veterinarian level. When the model was run without the outlying observations, the curvilinear shape of the association between CSQ score and depersonalization score was altered slightly to suggest lower CSQ scores associated with depersonalization scores in the mid-range, relative to the original model. The final model presented here includes the data from all 60 veterinarians and 800 clients.

Overall, the multilevel, multivariable model estimated a higher CSQ scores with veterinarian depersonalization in the upper half of the “high” category relative to lower depersonalization scores (Figure 3F). The model estimated a maximum difference of ~0.9 on the CSQ scale associated with veterinarian depersonalization scores in this population.

#### MBI-HSS Personal Accomplishment Subscale

The relationship between CSQ score and MBI-HSS personal accomplishment subscale score was linear (Table 2).

In multilevel, multivariable analysis, the association between CSQ score and veterinarian personal accomplishment score was confounded by the number of years the client had known the veterinarian. The interaction between veterinarian gender and age was significant in the model and reduced the magnitude and significance of the personal accomplishment coefficients. The full model had a significant fit relative to a null model (Wald χ^2^ = 44.00, *p* < 0.001), but the LRT was not significant (${\bar{\chi }}^{2}$ = 3.62, *p* = 0.16). The BLUPs had a normal distribution on graphical analysis with one outlier at the veterinarian level. When the model was run without the outlier, the magnitude and significance of the personal accomplishment score coefficient was reduced. The final model presented here includes the data from all 60 veterinarians and 954 clients.

Overall, the multilevel, multivariable model estimated a small but positive association between CSQ score and veterinarian personal accomplishment score (Figure 2B). The model estimated a maximum difference of ~0.4 on the CSQ scale associated with veterinarian personal accomplishment scores in this population.

#### ProQOL Burnout Subscale

The relationship between CSQ score and ProQOL burnout subscale score was not linear and a fourth-order polynomial provided the best fit (Table 2).

In multilevel, multivariable analysis, the association between CSQ score and veterinarian burnout score was confounded by the client income category. The interaction between veterinarian gender and age was significant in the model and reduced the magnitude of the association between CSQ score and burnout score. The full model had a significant fit relative to a null model (Wald χ^2^ = 49.73, *p* < 0.001), but the LRT was not significant (${\bar{\chi }}^{2}$ = 0.64, *p* = 0.73). The BLUPs had a relatively normal distribution on graphical analysis with one outlier at the veterinarian level. When the model was run without the outlier, the magnitude and significance of the burnout score coefficients was modestly reduced. The final model presented here includes the data from all 60 veterinarians and 840 clients.

Overall, the multilevel, multivariable model estimated relatively higher CSQ scores associated with mid-low and mid-high categories of burnout, and relatively lower CSQ scores in the average burnout category (Figure 3G). The model estimated a maximum difference of ~1.3 on the CSQ scale associated with veterinarian burnout scores in this population.

#### ProQOL Secondary Traumatic Stress Subscale

The relationship between CSQ score and ProQOL secondary traumatic stress subscale score was not linear and a fourth-order polynomial provided the best fit (Table 2).

In multilevel, multivariable analysis, the association between CSQ score and the secondary traumatic stress score was confounded by client ethnicity category and client income category. The interaction between veterinarian gender and age was significant in the model and modestly reduced the magnitude and significance of the secondary traumatic stress coefficients. The full model had a significant fit relative to a null model (Wald χ^2^ = 43.37, *p* < 0.001), but the LRT was not significant (${\bar{\chi }}^{2}$ = 2.13, *p* = 0.34). The BLUPs had a normal distribution on graphical analysis with three outliers at the veterinarian level. When the model was run without the outlying observations, the magnitude and significance of the secondary traumatic stress coefficients were reduced. The final model presented here includes the data from all 60 veterinarians and 823 clients.

Overall, the multilevel, multivariable model estimated relatively higher CSQ scores associated with veterinarian secondary traumatic stress scores in the average category or the upper half of the high category, with the greatest variation of CSQ scores associated with the highest secondary traumatic stress scores (Figure 3H). The model estimated a maximum difference of ~0.9 on the CSQ scale associated with veterinarian secondary traumatic stress scores in this population.

#### ProQOL Compassion Satisfaction Subscale

The relationship between CSQ score and ProQOL compassion satisfaction subscale score was not linear and a second-order polynomial provided the best fit (Table 2).

In multilevel, multivariable analysis, the association between CSQ score and compassion satisfaction score was confounded by veterinarian ethnicity category. The interaction between veterinarian gender and age was significant in the model and increased the magnitude and significance of the compassion satisfaction coefficients. The full model had a significant fit relative to a null model (Wald χ^2^ = 31.68, *p* < 0.001), but the LRT was not significant (${\bar{\chi }}^{2}$ = 2.04, *p* = 0.36). The BLUPs had a normal distribution on graphical analysis with one outlier at the veterinarian level. When the model was run without the outlying observations, the magnitude and significance of the compassion satisfaction score coefficients was modestly increased. The final model presented here includes the data from all 60 veterinarians and 995 clients.

Overall, the multilevel, multivariable model estimated an association between higher CSQ scores and compassion satisfaction scores in the high or low categories, relative to the average category (Figure 2C). The model estimated a maximum difference of ~0.6 on the CSQ scale associated with veterinarian compassion satisfaction scores in this population.

## Discussion

To our knowledge, this study is the first to assess the practical impact of veterinarian mental health on veterinary clients or patients. Overall, there were associations between client satisfaction scores and the mental health measures which were large enough in magnitude and significance to warrant close consideration of this relationship, but the complexity of the association may be the most important finding of this project. Ten of the eleven mental health measure scales and subscales demonstrated a polynomial, or curvilinear, relationship with client satisfaction, with the strongest associations demonstrated at the highest and/or lowest scores for most mental health measures, and weaker associations generally through the mid-range. At least five of the models estimated a difference in client satisfaction which has previously been associated with client adherence or non-adherence to veterinarian recommendations (8). Due to the high correlation between many of the mental health measures, these associations are not necessarily cumulative (i.e., the variation in client satisfaction score associated with anxiety cannot be added to the variation associated with burnout in a veterinarian experiencing both states), but suggest an impactful relationship between a veterinarian's mental wellbeing and the outcomes experienced by the veterinarian's clients. In turn, client satisfaction may influence client adherence (8) and business revenue (14) in the veterinary industry.

Of the associations modeled in this population, client satisfaction scores were estimated to have the most variation across the range of secondary traumatic stress scores relative to the other mental health measures, but the fourth order polynomial, curvilinear shape of the relationship suggests another, unmeasured variable may be confounding the association. Additionally, although the personal accomplishment scale demonstrated the hypothesized positive association with client satisfaction, the magnitude of the association was relatively small compared to many of the other scales, many of which estimated relatively higher client satisfaction scores at the end of the scale suggestive of poor mental health (for example, perceived stress, anxiety, emotional exhaustion, and depersonalization). These relationships were unexpected, given the more intuitive relationships reported in human healthcare, in which positive mental health measures have been commonly associated with relatively high patient satisfaction, and negative mental health measures with lower satisfaction. For example, Argentero et al. reported dialysis patient satisfaction had a positive association with the staff's level of personal accomplishment, and a negative association with the staff's level of emotional exhaustion (43). Physician depersonalization has also been negatively associated with patient satisfaction (16).

Causes for these associations have also been discussed in the literature on physicians, and are speculated to be due to physician verbal and non-verbal behaviors (44). A recent review of patient satisfaction in a healthcare context identified care provider attitude, and the reflection of this attitude in the provider's behavior, to be a key attribute of patient satisfaction (45). Examples included a provider's friendliness and approachability (45). Additionally, surgeons' tone of voice, including the level of anxiety or concern, was reported to be associated with a history of malpractice claims (46), which is also likely to reflect patient satisfaction. Future work on veterinary client satisfaction should consider measures of veterinarian verbal and non-verbal behaviors as possible predictors of client satisfaction which may affect the associations with mental health measures. Caregiver empathy, which has been studied repeatedly in non-veterinary contexts, may provide insight (47), but was not measured here.

Among physicians, empathy and empathic behaviors have a well-documented association with patient satisfaction (48), but are also hypothesized to increase the risk of burnout or compassion fatigue (47, 49, 50). This may result in an unexpected inverse association between caregiver mental health and client satisfaction. For example, in a study by Lafreniere et al., physicians with high levels of depersonalization were perceived by patients as more empathic (51). Veterinarian participants in this study with high levels of empathy may have had higher levels of client satisfaction, but also experienced a higher risk of poor mental health due to emotional labor. This may also explain the higher level of client satisfaction in veterinarians with high scores in anxiety, emotional exhaustion, and depersonalization.

Alternatively, there may have been an insufficient sample size in some of the “high” categories, possibly due to introduction of an unforeseen selection bias during the recruitment of veterinarians (i.e., veterinarians who participated in this study were less likely to score “high” in the negative mental health measures than veterinarians who participated in a recent Canada-wide survey, on which sample size calculations were based), resulting in insufficient power. This may be corroborated by the relatively large confidence intervals at the upper and lower ends of each mental health measure in this population. The sample size for veterinarians was calculated a priori, with anticipated variation in mental health measures based on the results of a recent Canada-wide survey (1). However, the mean mental health scores for participating veterinarians in this study trended toward the “well” end of the larger survey range (1). It is likely that veterinarians with high levels of burnout or other negative mental health measures declined participation in this study, while those with high levels of positive measures, such as personal accomplishment, were more likely to agree to participate. This is reflected in the responses received from non-participants, such as “feeling too busy.”

In contrast with the negative mental health measures, veterinarian compassion satisfaction appeared to have a negative relationship with client satisfaction through much of the score range, with a positive association only in the “high” category of compassion satisfaction. Recent research into sources of stress in the veterinary profession has drawn attention to ethical conflict in the veterinarian-client-patient relationship, highlighting the importance of differentiating between the veterinarian-patient relationship and the veterinarian-client relationship (52). It is possible, for example, that while veterinarians derive satisfaction from empathy toward their animal patients, client satisfaction is more reliant on expressions of empathy directed at the client (53). Further studies may focus on distinguishing veterinarian feelings of compassion satisfaction and empathy originating from their treatment of animal patients, and those feelings derived from their work with human clients.

The interaction between veterinarian age and gender emerged as an important factor in client satisfaction, as anticipated, and frequently impacted the magnitude of the association between client satisfaction the mental health measure. Veterinarian age appeared to have a positive association with client satisfaction for female veterinarians, but a negative one for male veterinarians. It is important to note that the number of female veterinarian participants was greater than males, resulting in the wider confidence intervals seen for males. Research on the physician-patient dyad suggest that caregiver gender may impact both caregiver behavior (54, 55) and patient expectations (38). Female physicians are more likely to communicate a high degree of empathy (54, 55), and their patients' satisfaction is more dependent on this demonstration of empathy (38). Similar results have been found among veterinarians (56). It is interesting to note that although the caregiver-recipient gender dyad has been a significant factor in previous research on both physicians and veterinarians (38, 56), the client gender was not significant in this study.

In human medicine, age of physician may be perceived as experience or skill, and is associated with higher patient satisfaction (37). Further, the age of a physician appears to mediate the negative association between burnout and reduced professionalism, which is strongest among young physicians (6). Similarly to gender, it is likely that the age of the veterinarian impacts both the veterinarian's behavior, and client expectations of veterinarian behavior and communication style. More experienced veterinarians may also experience less mental and emotional impact based on the satisfaction or dissatisfaction of their clients, relative to less experienced veterinarians.

Client demographics and appointment-specific variables had little impact on the magnitude of the association between veterinarian mental health measures and client satisfaction, despite significant associations with CSQ scores during univariable analysis in the present study, and in a previously published study (11). Client income impacted the relationship between CSQ score and veterinarian mental health measure in seven of eleven models, and client education, in three of eleven models. Findings from human healthcare studies suggest aspects of provider-recipient communication are dependent on perceived social gap (57). Patients with higher education levels appear to place greater value in patient-centered care (58), tend to be more active participants in their own care (59), and may perceive a smaller social gap between themselves and their physician (57). Conversely, patients that perceive themselves to be of a lower socioeconomic status than the physician may be less likely to pose questions and participate in treatment decisions (57).

Client ethnicity impacted the relationship between client satisfaction and veterinarian mental health measure in three of eleven models, and veterinarian ethnicity in one of eleven models. Ethnic and racial differences have also been reported in previous research on caregiver-recipient interactions (57). Typically, differential healthcare provision to minorities has been associated with biases and stereotypes, with communication style being particularly impacted (57). Given the instrument used in the present study measures perception, it is also possible that cultural differences impacted client expectation and evaluation of their interaction with the veterinarian. Future studies would be helpful to further explore ethnic or cultural differences in veterinarian-client communication.

The number of years the client had known the veterinarian had a strong relationship with client satisfaction in univariable analysis and impacted the association with veterinarian mental health in three of the models. Several possible explanations may exist: clients who have not changed veterinary care providers for many years may be inherently more easily satisfied; clients with a prior relationship with the veterinarian may be more “forgiving” of a less satisfying encounter [the ‘halo effect’; (60)]; clients with many prior interactions with a veterinarian may have based their questionnaire responses on a more generalized view of the veterinarian, rather than on the specific appointment; veterinarians interacting with long-term clients may behave or communicate differently with that client than with a new client; and veterinarians who are satisfied with their clinic environment may be less likely to seek employment elsewhere, and thus are more likely to develop long-term relationships with clients. The human physician-patient relationship and continuity of care are well described in the literature as important factors in patient satisfaction (61). It is worth noting that the variable for the length of the veterinarian-client relationship was not highly correlated with the age of the veterinarian, and they appeared to act independently in the models.

Because the present study was cross-sectional, the direction of causal relationships cannot be determined. Hence, the associations described here may suggest that overall client satisfaction has a significant impact on veterinarian mental health, which may be explained by the dyadic nature of the relationship between the veterinarian and the client. Among physicians, several aspects of the physician-patient relationship have been associated with physician satisfaction with office visits, including the number of prior visits with the same patient (62). Similarly, veterinarians' satisfaction with the veterinarian-client-patient relationship predicts their global satisfaction with an appointment (63). Building relationships with clients and patients has been shown to contribute to compassion satisfaction, but also compassion fatigue (64), which may, in part, explain why the relationship between veterinarian mental health and client satisfaction is so complex. A cohort study would further clarify this relationship.

The practical importance of the difference in CSQ scores demonstrated in this study should be interpreted with caution. Although one previous study reported an association between CSQ scores and client adherence to veterinarian surgical or dental recommendations (8), this remains a novel area of research in veterinary medicine. Further studies are needed to explore the association between client satisfaction and client adherence to other types of veterinarian recommendations, veterinary clinic revenue, and other effects suggested by the human medical literature.

Previous work involving the CSQ score has suggested that only 7.5% of the variation in client satisfaction is determined at the veterinarian level (11), suggesting that if client satisfaction impacts veterinarian mental health, clinic-level interventions to improve client satisfaction would have the potential to positively impact veterinarian mental health. In this case, the clinic may have a valuable role to play in improving veterinarian mental health by instituting practices which may increase client satisfaction. For example, the full integration of paraprofessionals as members of the healthcare team has been suggested to improve service quality (65). Nurse practitioners can play an important role in a primary care setting, providing care that is equivalent to that of physicians, in many instances (66); veterinary technicians could equally take on a larger role in client communication, education, and consultation.

An indication of possible selection bias was that participants had less variation in mental health measures, and an overall higher level of mental health, than anticipated. A study population with more diverse mental health may provide further insight, especially given the non-linear association between client satisfaction and many of the mental health measures. Additionally, bias may have been introduced as veterinarians who felt they had a positive relationship with their clients may have been more likely to participate, though the range of CSQ scores was similar to that of a previous study with this scale, which was also voluntary and thus susceptible to the same bias (11). The association of veterinarian mental health measures to client satisfaction may thus be underestimated in this population. The high level of skew and low level of variation present in CSQ scores was as expected (11), though previous analysis of the scale has suggested that as many as sixty-one clients per veterinarian may be needed to accurately estimate clients' satisfaction with a veterinarians (11), a number which was not logistically feasible within this study. Future work may also standardize or control for the length of time between the appointment and the completion of the client satisfaction survey. Results from human medicine suggest that immediate post-appointment patient satisfaction is based primarily on communication and interaction quality, while patient satisfaction surveys completed at a later date will incorporate satisfaction with health outcomes for the patient (17), and may have a lower scores on average than those done in-clinic (54). Thus, client satisfaction surveys not done immediately following the appointment may have had a relatively reduced association with veterinarian mental health measures.

Most small animal veterinary clinics rely heavily on a small business model and client satisfaction is an important determinant of client loyalty and referrals in the medical industry (67). Given the relatively small variation in client satisfaction scores overall [and the potential for further impacts on client adherence (8), veterinarian and clinic income (14), and veterinarian career satisfaction (15)], the significant associations with veterinarian mental health reported here warrant further investigation.

## Data Availability Statement

The datasets generated for this article are not readily available because of ethical restrictions. Requests to access the datasets should be directed to Dr. Andria Jones-Bitton (email: aqjones@uoguelph.ca).

## Ethics Statement

This study was approved by the Research Ethics Board of the University of Guelph (REB #17-08-009) and written informed consent was obtained from all veterinarians and veterinary clients prior to their participation, in accordance with the Declaration of Helsinki.

## Author Contributions

CB, JC, DK, and AJ-B conceived of the study, and all authors contributed to the study design. CB, AJ-B, and DK secured funding. Project administration was handled by AJ-B. JP recruited participants and collected data. All authors contributed to data analysis and interpretation. JP wrote the first draft of the manuscript, and all authors contributed to manuscript revision and approved the submitted version.

### Conflict of Interest

The Ontario Veterinary College funded JP's personal stipend, and the gift from Zoetis Canada funded CB's stipend. JC regularly receives research grants, consults for and receives honoraria from various veterinary organizations and commercial companies including Zoetis Canada and Royal Canin. The remaining authors declare that the research was conducted in the absence of any commercial or financial relationships that could be construed as a potential conflict of interest.

## Abbreviations

BLUP: Best linear unbiased predictor
CD-RISC: Connor-Davidson Resilience Scale
CI: Confidence interval
CSQ: Client Satisfaction Questionnaire
HADS: Hospital Anxiety and Depression Scale
LRT: Likelihood ratio test
MBI-HSS: Maslach Burnout Inventory—Human Services Survey
ProQOL: Professional Quality of Life Scale
PSS: Perceived Stress Scale.
