# Corrigendum: The Complex Relationship Between Veterinarian Mental Health and Client Satisfaction

**DOI:** 10.3389/fvets.2020.616608

**Published:** 2020-11-24

**Authors:** Jennifer L. Perret, Colleen O. Best, Jason B. Coe, Amy L. Greer, Deep K. Khosa, Andria Jones-Bitton

**Affiliations:** Department of Population Medicine, Ontario Veterinary College, University of Guelph, Guelph, ON, Canada

**Keywords:** veterinarian, mental health, client satisfaction, veterinarian-client-patient relationship, burnout, compassion fatigue, resilience, stress

In the original article, there was an error. Specifically, the Data Availability Statement was incorrect.

A correction has been made to the Data Availability Statement. The correct statement should read:

The datasets generated for this article are not readily available because of ethical restrictions. Requests to access the datasets should be directed to Dr. Andria Jones-Bitton (email: aqjones@uoguelph.ca).

The authors apologize for this error and state that this does not change the scientific conclusions of the article in any way. The original article has been updated.

